# Exploring the challenges in leadership roles experienced by nurse managers in a mining primary healthcare setting in South Africa

**DOI:** 10.4102/curationis.v44i1.2196

**Published:** 2021-08-10

**Authors:** Sanele E. Nene

**Affiliations:** 1 Department of Nursing, Faculty of Health Sciences, University of Johannesburg, Johannesburg, South Africa

**Keywords:** leadership roles, challenges, experiences, nurse managers, primary healthcare

## Abstract

**Background:**

The challenges in leadership roles hinder the rendering of quality primary healthcare service in the mines. Mining, the heart of the South African economy, requires good health to its personnel to carry out operations. However, nurse managers, the leaders in a mining primary healthcare setting experience difficulties in their leadership roles.

**Objectives:**

The aim of this study was to explore and describe the challenges in leadership roles experienced by nurse managers in a mining primary healthcare setting in South Africa.

**Method:**

The study was conducted in a mining primary healthcare setting in West Rand, Gauteng province, South Africa. A qualitative, exploratory, descriptive design that is contextual in nature, using a phenomenological approach, was adopted. Data from nurse managers in the mine were collected and data saturation was reached by the seventh participant. The study followed Giorgi’s four stages of the phenomenological descriptive data analysis. An expert independent coder in qualitative research coded the data, and consensus on the findings was reached with the researcher.

**Results:**

Three subthemes emerged from the study: mining management and unions interfere with nurse managers’ leadership roles, incongruent mining primary healthcare policies and communication gap between nurse managers and mining management.

**Conclusion:**

The triangulation of nurse managers, mining management and unions requires a collective fusion to directly tackle the challenges in leadership roles in mining primary healthcare.

## Introduction

Challenges in leadership roles affect the quality of primary healthcare service rendered in the mining industry (Britton 2020; Du Plessis 2018; Wohlgemuth 2017). The mining industry is the heart of the South African economy, contributing more than R351 billion to the South African gross domestic product (GDP) annually (Abuya 2018; Minerals Council South Africa 2020). It is therefore essential to ensure that health problems of mine workers that are at primary healthcare level are managed in a mining primary healthcare setting (South African National Health Insurance Bill 2019).

Nurse managers are part of mine workers who are employed to lead in a mining primary healthcare setting. If health problems of mine workers are not managed at primary healthcare level, this will result in an increase in hospital stay and long sick leaves, and the worker’s performance then will be affected, thus reducing the mining production. The management role of a nurse manager in a primary healthcare setting is to ensure that the resources required to execute daily activities are available, safe and fairly distributed. There are challenges in leadership roles experienced by nurse managers that affect the effectiveness of their leadership. The leadership role of a nurse manager is to inspire and empower the followers during the execution of daily activities in order to achieve a specific goal (Al-Dossary 2017). A good manager ought to have good leadership and management skills, to be a good leader (Lamker 2019; Skeepers & Mbohwa 2015).

Leadership roles direct nurse managers to focus on collectively achieving the vision of a mining primary healthcare setting (Jooste & Hamani 2017; Kgatle 2018; Steinhouse 2018). Nurse managers execute leadership roles daily in their mining primary healthcare work life to meet the health needs of mine workers. Nurse managers facilitate their leadership roles by developing constructive relationships with all mining stakeholders, which create professional bonds to achieve collective goals, and benefit patient, and the mining primary healthcare setting (Vasconcelos et al. 2017). The mining stakeholders are mine workers, unions, mining management and regulatory bodies such as the Department of Health and the Department of Minerals Resources (Chamber of Mines, South Africa 2016; Nene, Ally & Nkosi 2020).

Nurse managers face challenges in leadership roles in a mining primary healthcare setting in South Africa. Banaeianjahromi and Smolander (2017) and Nene et al. (2020) confirmed that these challenges experienced by nurse managers in a mining primary healthcare setting include lack of effective communication, bureaucratic mining management and union influence. Primary healthcare is a frontline service that manages the health problems at this level for all the mine workers. The Alma Ata Model of primary healthcare elicits that health problems of mine workers must be tackled at primary healthcare level by providing health promotive, preventive, curative and rehabilitative services accordingly (Ata 1978; Gillam 2008).

Leadership roles in a primary healthcare setting assist nurse managers to facilitate the process of ensuring that health users receive appropriate assessment, treatment and care timely (Booysen, Jooste & Sibiya 2018; South African Department of Health, National Health Insurance Bill 2019; United Nations 2019). The World Health Organization (WHO) (2019) reported that exploring the challenges in leadership roles in a mining primary healthcare setting is essential to achieve the Sustainable Development Goal (SDG) number 3. This SDG addresses the fact of ensuring good health for all, including the mine workers.

The South African Department of Health is transitioning to National Health Insurance to mitigate the challenges in leadership roles (South African National Health Insurance Bill 2019). The National Health Insurance will strengthen the healthcare system by re-engineering primary healthcare to improve access to healthcare for the population while reducing the burden of diseases (Girdwood et al. 2020; Mofolo, Heunis & Kigozi 2019; South African National Health Insurance Bill 2019). In a mining primary healthcare setting, improved access to healthcare and good health is demonstrated by treating mine workers at primary healthcare level to prevent complications that might lead to longer hospitalisation. Hence, this ensures that mine workers are fit to work and are productive, which yield to job sustainability and economic growth.

The activities mentioned in the statement above will contribute to ensure that the SDG number 8 – ‘decent work and economic growth’ – is achieved (WHO 2019). Nurse managers in a mining primary healthcare setting are obligated to maximise the quality of services rendered as the foundation of the health system (South African National Health Insurance Bill 2019).

The challenges in leadership roles in primary healthcare can be resolved by transferring enthusiasm, empowerment, assertiveness and building relationships (Jooste 2017). Identifying and resolving challenges in leadership roles ensures that accessible primary healthcare services are rendered to the mine workers (Froneman 2017). An environment that is conducive for the delivery of quality healthcare is created by tackling challenges in leadership roles collectively with all stakeholders. Nurse managers should be supported with all the resources required to achieve their leadership goals at all levels of the organisation so that they are able to resolve operational challenges (Mbebwo 2019; Mofolo et al. 2019:1).

Mining primary healthcare setting has its own leadership roles dynamics and it includes dealing with different stakeholders simultaneously to ensure that the objectives of the organisation are achieved. The collective objectives ensure that mine workers are healthy, safe and productive to sustain the business, and thus contribute significantly to the economy of the country (Froneman 2017).

In their studies in mining primary healthcare settings, Jooste and Hamani (2017) and Balfour (2018) concluded that there should be a shift in how health services are rendered in a mining industry, to ensure that challenges and demands are collectively confronted. This shift requires nurse managers to be good role models and to understand their leadership roles. The leadership roles of nurse managers close the gap in primary healthcare by ensuring that the majority of health problems are diagnosed and treated at this level (South African National Health Insurance Bill 2019). Against this background, the researcher sought to explore and describe the challenges in leadership roles experienced by nurse managers in a mining primary healthcare setting in South Africa.

From the background, it is clear that nurse managers are leaders in a mining primary healthcare setting. However, in a specific mining primary healthcare setting in South Africa, the researcher has observed that the nurse managers experience challenges in their leadership roles. On execution of leadership roles, nurse managers experience the interference from the mining management and unions. Nurse managers are told what to do and how to do it, rather than being allowed and emancipated to lead as experts in primary healthcare. It is not the obligation of the mining management and unions to lead in a primary healthcare setting as it requires specific operational skills that they do not have (The South African Human Resource for Health, 2012/2013–2016/2017). Sherman (2012), Zydziunaite and Souminen (2014) and Liphadzi, Aigbavboa and Thwala (2016) found the same results that nurse managers are expected to facilitate their leadership roles, while, on the other hand, they are dealing with a high pressure from the management that interfere with their daily activities. The regulation for the course leading to Diploma in Nursing Administration (South African Department of Health, Regulation 2554 of 1985 as amended) stipulates that nurse managers can lead in a healthcare establishment, provided that they have a nursing management qualification. Hence, all the nurse managers of this mining primary healthcare setting have obtained a nursing management qualification. Amid this, the researcher was prompted to the following research question: what are the leadership roles’ challenges experienced by nurse managers in a mining primary healthcare setting?

## Research methods and design

The purpose of this study was to explore and describe the challenges in leadership roles experienced by nurse managers in a mining primary healthcare setting in South Africa.

### Setting

The study was conducted in a mining primary healthcare setting in West Rand, Gauteng province. West Rand is a gold and uranium mining area, and primary healthcare services are offered in house by some of the mining organisations, and hence nurse managers are mine employees. In this mining organisation, comprehensive primary healthcare services are provided. These services include primary healthcare emergencies, tuberculosis management, human immunodeficiency virus and acquired immunodeficiency syndrome (HIV and AIDS) management, family planning, and medical and surgical services managed at primary healthcare level. Nurse managers are responsible for eight mining primary healthcare clinics. There are 15 nurse managers employed in this mining primary healthcare setting, with about 45 nursing staff members reporting to them, providing services to about 17 000 mine workers.

### Method

A qualitative, exploratory, descriptive design that is contextual in nature, using a descriptive phenomenological approach, was adopted in this study to explore and describe the challenges in leadership roles experienced by nurse managers in a mining primary healthcare setting in South Africa (Creswell & Creswell 2018).

### Population and sampling

The target population in this study were nurse managers from a mining primary healthcare setting in West Rand, Gauteng province, South Africa. These nurse managers were selected because they were more informative about the phenomena and were willing to share their experiences (Polit & Beck 2018). The participants were selected following a purposive sampling method. Data saturation were reached by the seventh participant (Creswell & Creswell 2018).

#### Inclusion and exclusion criteria

Inclusion criteria for this study were as follows: nurse managers working in this mining primary healthcare setting with more than one year of experience and willing to participate. These nurse managers had to be registered with the South African Nursing Council as professional nurses and nurse administrators. Nurse managers with less than one year of experience in a mining primary healthcare setting, and who were not working in primary healthcare such as occupational health nurse managers, were excluded from the study.

### Data collection

In-depth individual descriptive phenomenological, semi-structured interviews were conducted to collect data from the participants in their workplace between September and December 2017. The information session on the study was held to recruit the participants, and the consent forms to participate in the study and of audio recordings were completed afterwards by the participants who were willing to participate. Arrangements on time, date and venue for the interviews were made with the participants according to their work schedule and interviews were held in their offices. The researcher was a case manager in this mining primary healthcare service during the study, reporting to the head of case management. The head of case management was reporting to the nurse managers, and, therefore, there was no close relationship between the researcher and the participants. The experiences of the participants were transcribed as described by them (Gray, Grove & Sutherland 2017). The researcher conducted the interviews and took the field notes during the interviews to make an accurate description of emotions and gestures as expressed by the participants. Probing, paraphrasing and reflecting were amongst the facilitative communication techniques adopted by the researcher during data collection. The individual descriptive phenomenological semi-structured interviews lasted for about 30–45 min with each participant.

### Data analysis

Giorgi’s four stages of the descriptive phenomenological data analysis were employed in this study (Holloway & Gavin 2017). The researcher and the independent coder independently engaged with the verbatim transcripts, field notes and audio recordings to develop the categories that formed themes of the study. The descriptive words were used to categorise the themes into topics and sub-topics. Meaningful consistent statements regarding the participants’ experiences were made by the researcher and confirmed by the independent coder. The independent coder who coded the data is an expert in qualitative research. Consensus on the findings was reached in the verification meeting held with the independent coder.

### Trustworthiness

Lincoln, Lynham and Guba’s (2011) model on trustworthiness was applied in this study. Credibility, dependability, transferability and confirmability, which are the four strategies of trustworthiness, were applied throughout the study. Credibility was ensured through triangulation using more than one method of data collection. Dense data description was conducted to maintain the dependability of the study. Integration of data with the recent relevant literature was effected to ensure transferability. The research process was closely monitored by the research supervisors who were experts in qualitative research to maintain accuracy, relevancy and confirmability of the study.

### Ethical considerations

Dhai and McQuoid-Mason’s (2011) ethical principles of autonomy, beneficence, non-maleficence and justice guided this study. The Faculty of Health Sciences Research Ethics Committee of the University of Johannesburg, located in Gauteng, Johannesburg, provided ethical clearance for the study (clearance number: REC-01-73-2017). The management of a mining primary healthcare setting granted permission to conduct the study. An informative session on the study was held with the participants and they were informed that the findings of the study will be shared with them to improve practice in primary healthcare. During the informative session, the research question was also discussed with the participants so that the question is not a surprise to them on the day of the interview.

## Results

Seven participants were interviewed, and the findings that emanated are presented below. The demographics of the participants are presented in Box 1.

BOX 1 Participants’ demographics.

**Table:** 

*N*	7
Race	White 1, African 6
Gender	Women 3, Men 4
Age range	38–60 years

Three themes emerged as challenges in leadership roles experienced by the nurse managers, namely, mining management and unions interfere with nurse managers’ leadership roles, incongruent mining primary healthcare policies and communication gap between the nurse managers and mining management.

### Mining management and unions interfere with nurse managers’ leadership roles

The participants mentioned that the mining management and unions interfere with the execution of their leadership roles, and their activities are influenced by the management and unions. This statement is confirmed by the following quotations from the participants:

‘You know one experience that the mining management don’t get informed from the operations to say these are the challenges that we sit with; they just sit at the strategic level making decisions for us, interfering with primary healthcare activities.’ [*Taking a deep breath*]. (Participant 4, Male, 38 years)

‘You also experience a challenge of dealing with the demanding unions, who are interfering with what is happening at primary healthcare.’ (Participant 7, Male, 59 years)

Another participant mentioned that:

‘I think because we [*are*] also working with a highly unionized environment, so it put[*s*] you in a very difficult situation when you have to make primary healthcare decisions, because if the union is saying I don’t agree with one and two, you can’t implement.’ [*Emotional, tone of voice shaking*]. (Participant 5, Female, 40 years)

### Incongruent mining primary healthcare policies

Most of the participants mentioned that their experiences involve the challenge of using mining primary healthcare policies that are outdated, and not relevant for the practice. Confirming this are the following quotations from the participants:

‘My experience is that we have to use old policies that are no longer relevant for the setting.’ (Participant 2, Female, 60 years)

‘You are on board leading, but the policy is not recognizing you and it is irrelevant for the practice.’ (Participant 3, Female, 57 years)

One participant alluded that:

‘Our biggest challenge are the old rules and policies that govern you … and we need to function within them.’ [*Using both hands to explain*] (Participant 6, Female, 43 years)

### Communication gap between the nurse managers and mining management

Nurse managers alluded that there is a challenge of communication gap that affects the execution of their leadership roles. The communication gap is between the nurse managers and mining management. Other participants mentioned that there is a lack of open and honest communication in mining primary healthcare setting. This is affirmed by the following quotations:

‘I think a bit of gap that we have at this stage that we experience as a challenge, it’s communication.’ (Participant 2, Female, 60 years)

‘I have experienced that there is no open and honest communication here.’ [*Putting the left hand in the left cheek*]. (Participant 4, Male, 38 years)

Another participant echoed that they experience absence of equity in communication sharing and that information is also disclosed to them at a later stage:

‘You get that the person you report to didn’t disclose everything, and they only disclose it at a very late stage.’ [*Using both hands to explain, looking worried in the face*]. (Participant 7, Female, 59 years)

## Discussion of results

### Mining management and unions interfere with nurse managers’ leadership roles

The findings of this study revealed that one of the challenges experienced is that the mining management and unions interfere with the nurse managers’ leadership roles. The mining management should avoid interfering with operational activities by ensuring that the strategic goals are congruent to the operational goals (Jooste 2017). Spehar et al. (2017) posit that the mining management, unions and nurse managers should collectively work as a team, to deal with the challenges experienced at primary healthcare. The purpose of mining management and unions in primary healthcare should be to support nurse managers to promote best practices that will maintain a healthy workforce (Abuya 2018). Dorigatti (2017) argued that unions should not interfere with the leadership roles of management, but should channel their focus to challenging and improving the working conditions of the mine workers.

Furthermore, it was also noted by the participants that they experience a challenge of a mining environment that is highly unionised and that the unions are demanding, instead of engaging. Unions should not be demanding towards nurse managers but instead they should engage on developing a culture of facilitating effective corporate interaction to manage leadership roles challenges in a mining primary healthcare setting (Fortunato, Gigliotti & Rubben 2017). Crossler et al. (2017) attest that the demanding attitudes of the unions portray a picture of being perceived as problematic stakeholders, and as a challenge rather than a problem solver (Holgate 2015; Wohlgemuth 2017).

### Incongruent mining primary healthcare policies

Nurse managers also alluded that they experience a challenge of incongruent mining primary healthcare policies. The available policies are old and no longer relevant for the mining primary healthcare practice today, and hence these policies do not recognise nurse managers as leaders. Lawlis, Knox and Jamieson (2016) stated that the mining management should not only consider policy compliance, but they should also determine if the policy is still relevant for the practice. Natera, Tomassini and Vera-Cruz (2019) asserted that policies continuously serve the purpose when they are being updated to suit the demands and the dynamics of the practice. Nurse managers are expected to comply with outdated policies that are not congruent to the *status quo*, and this delays the achievement of operational objectives, deeming leaders as ineffective (Lawlis et al. 2016; Sechko & Romanova 2017).

Other participants alluded that mining primary healthcare policies governing them are old and do not recognise them as leaders. Leaders who are not recognised by the existing policies that are guiding the practice feel devalued and discouraged, and this affects the performance of the organisation (Veronesi, Kirkpatrick & Altanlar 2019). Tingvoll, Saeterstrand and McClusky (2016) and Masters (2017) reported that leadership roles are increasing and cannot be achieved using inherent policies that cannot be challenged, and that is not recognising all the relevant stakeholders. The leadership role of a nurse manager in primary healthcare is to develop and update policies to ensure relevance in practice and to eradicate role ambiguity and delays that may arise from the use of incongruent policies (Jooste 2017; Nene et al. 2020). Nurcahyo et al. (2018) attest that the use of incongruent policies is a challenge, because they inhibit the engagement of nurse managers for the success of the organisation. The current practice and policies governing the mining primary healthcare should be reviewed and updated to ensure that they remain effective and relevant (Mutatina et al. 2017).

### Communication gap between the nurse managers and mining management

Nurse managers posit that there is a communication gap between them and the mining management, and this gap affects the execution of their leadership roles. Communication gap makes it difficult to set common goals and achieve a shared understanding, and this put the organisation at risk of personnel’s distrust, lack of innovation and loss of competitive edge (Banaeianjahromi & Smolander, 2017). This confirms that effective communication is the engine propelling the organisation towards reaching its vision. Peiter, De Melo Lanzoni and De Oliveira (2016) alluded that the challenge of a communication gap affects clear descriptions of leadership roles in organisations, making work overload unavoidable. Napier et al. (2018) confirmed that closing a communication gap is essential to the success of any leadership information-sharing initiative and framework of the organisation. Communication gap makes it difficult for nurse managers to execute leadership roles, and it can be interpreted as a river that the mining management, unions and nurse managers have to cross collectively as a team.

Participants further stated that there is no open and honest communication in the mining primary healthcare setting, and that communication of information is not released simultaneously to all stakeholders. The union members receive information before the nurse managers receive it from the mining management; by the time nurse managers are discussing the issues with the nursing personnel, these issues are already known to the nursing personnel. Woodward, More and Van Der Heyden (2016) elicited that releasing of information simultaneously to all relevant stakeholders assists to address communication challenges such as lack of open and honest communication. Griffiths (2017), Napier et al. (2018) and Yusof et al. (2018) confirmed this by corroborating that mining leaders should develop an environment that is conducive for open and honest communication by sharing the critical information with all relevant stakeholders timely and simultaneously. Closing a communication gap collectively will lead to the successful creation of a therapeutic working environment, where all stakeholders feel valued, appreciated and automatically retained.

## Limitations

This study is contextual in nature, limiting the generalisation of the study to other mining primary healthcare services. The researcher experienced that there is limited literature on mining primary healthcare, and it was therefore challenging to provide dense description of data within this setting for integration with relevant literature. Some of the participants were scared to provide more details on their leadership roles’ experiences in the mining primary healthcare setting.

## Implications of the study

Nurse managers in mining primary healthcare settings can implement the findings of this study to approach and resolve their leadership roles challenges. The challenges identified from this study can be used for the personal and professional development of nurse managers in mining primary healthcare settings.

## Conclusion

Nurse managers are leaders in a mining primary healthcare setting but they are experiencing challenges in leadership roles. The mining management and unions are interfering with their leadership roles, and existing primary healthcare policies are incongruent to the leadership roles of nurse managers. The other challenge is that there is a communication gap between the nurse managers and mining management.

## Recommendations

Challenges in leadership roles experienced by nurse managers should be identified and resolved collectively by all the relevant stakeholders in a mining primary healthcare setting to improve practice (Jooste 2017). The mining management and unions should recognise and trust nurse managers as leaders in primary healthcare to enable them to execute their leadership roles without interference. The recognition of nurse managers as leaders will afford them with an opportunity to update the existing policies and to critique these policies for congruence to ensure best practice, and to close the communication gap in healthcare. The challenges in leadership roles that emanated from this study should be collectively addressed by nurse managers, mining management and union leaders as key stakeholders in a mining primary healthcare setting. Quantitative research to evaluate the leadership challenges of nurse managers in mining primary healthcare should be conducted.
